# Elevated Level of Troponin but Not N-Terminal Probrain Natriuretic Peptide Is Associated with Increased Risk of Sudden Cardiac Death in Hypertrophic Cardiomyopathy Calculated According to the ESC Guidelines 2014

**DOI:** 10.1155/2017/9417908

**Published:** 2017-11-20

**Authors:** Renata Rajtar-Salwa, Rafał Hładij, Paweł Petkow Dimitrow

**Affiliations:** 2nd Department of Cardiology, Jagiellonian University Medical College, Kraków, Poland

## Abstract

The aim of this study was to assess the relationship between biomarkers (high-sensitive troponin I [hs-TnI], N-Terminal probrain natriuretic peptide [NT-proBNP]) and calculated 5-year percentage risk score of sudden cardiac death (SCD) in hypertrophic cardiomyopathy (HCM). *Methods*. In 46 HCM patients (mean age 39 ± 7 years, 24 males and 22 females), echocardiographic examination, including the stimulating maneuvers to provoke maximized LVOT gradient, had been performed and next ECG Holter was immediately started. After 24 hours, the ECG Holter was finished and the hs-TnI and NT-proBNP have been measured. Patients were divided according to 1/value of both biomarkers (hs-TnI-positive and hs-TnI-negative subgroups) and 2/(NT-proBNP lower and higher subgroup divided by median). *Results*. In comparison between 19 patients (hs-TnI positive) versus 27 patients (hs-TnI negative), the calculated 5-year percentage risk of SCD in HCM was significantly greater (6.38 ± 4.17% versus 3.81 ± 3.23%, *P* < 0.05). In comparison between higher NT-proBNP versus lower NT-proBNP subgroups, the calculated 5-year percentage risk of SCD in HCM was not significantly greater (5.18 ± 3.63% versus 4.14 ± 4.18%, *P* > 0.05). *Conclusions*. Patients with HCM and positive hs-TnI test have a higher risk of SCD estimated according to SCD calculator recommended by the ESC Guidelines 2014 than patients with negative hs-TnI test.

## 1. Introduction

The risk factors of sudden cardiac death (SCD) for hypertrophic cardiomyopathy (HCM) in the ESC Guidelines [1] included echocardiogram, electrocardiographic (ECG) Holter monitoring, and clinical variables. The calculator for sudden cardiac death risk [1] has not included any biomarker. Recently, Kehl et al. [2] reviewed the available data regarding the usefulness of natriuretic peptides and troponins in HCM. Concentrations of natriuretic peptides, and to a lesser extent of troponins, correlate with left ventricular thickness, symptom status, and left ventricular hemodynamics by Doppler measurements (left ventricular filling pressure, left ventricular outflow tract gradient).

Neither ischemic biomarker nor signs and symptoms of myocardial ischemia are included in the calculator [1]. However, ischemic response to stress revealed by echocardiographic methods becomes important prognostic player [3, 4].

Currently used high-sensitivity troponin I (hs-TnI) is an super precise biomarker for the detection of myocardial ischemia. In previous HCM studies, measurements of hs-Tn were only at a resting (without stress in unnatural condition) echocardiography and not timely synchronized with maneuvers to provoke LVOTG by natural stimuli reflecting daily common physical activity for patients [5–8]. Moreover, measurements of hs-Tn were also not timely synchronized with the Holter monitoring. So far, we have used the following protocol: 24-hour cycle—8 a.m., echocardiography with LVOTG provocation by natural stimuli (orthostatic test and Valsalva test [1, 9–13]; the observation was divided into 2 periods: day phase physical activity with probable episodes of provocable LVOTG (unmeasurable) and night phase period as a potential time for rise of troponin, in which the level has been measured after night at 8 a.m. in the next day). Between echocardiography and biomarker sampling, 24-hour Holter electrocardiography (ECG) was recorded and then the measurement of hs-TnI (the biomarker level has a close temporal relationship with findings on Holter ECG). This protocol seems to be reasonable because hs-TnI levels may be a potential cause of life-threatening ventricular arrhythmias occurring during the previous 24 hours.

The aim of this study was to assess the relationship between biomarker concentrations (hs-TnI, NT-proBNP) and calculated 5-year percentage risk score of SCD in HCM.

## 2. Methods

Consecutive patients with HCM were recruited to the study. Informed consent was obtained from each participant. All patients fulfilled conventional diagnostic criteria for HCM. The criteria for diagnosis of HCM, according to the ESC Guidelines, were the presence of left ventricular (LV) wall thickness of at least 15 mm without any other cause that could lead to ventricular hypertrophy [1, 13]. The exclusion criteria were as follows: sport activity more than recreational, prior myocardial infarction, current symptoms suggestive of coronary artery disease, concomitant neoplasm, infection, or renal failure. Subjects who had a history of alcohol septal ablation or septal myectomy were not included into the present study.

The final sample included 46 patients with HCM (mean ± SD age, 39 ± 7 years; 24 men and 22 women).

Patients on current pharmacotherapy were studied according to the abovementioned protocol. Patients have been asked to perform their common day physical activity and nocturnal resting. This protocol seems to be reasonable because hs-TnI levels may be related with labile, dynamic nature of LVOTG with fluctuating peaks in daytime (provoked LVOTG as a potential cause of myocardial ischemia).

### 2.1. First Model of Risk Calculation (Only the Current ECG Holter)

For calculating the percentage value of HCM risk score SCD, we assessed the following parameters: episodes of nonsustained ventricular tachycardia (nsVT) in current Holter monitoring (defined as three or more consecutive ventricular beats > 120 beats per minute) and two-dimensional (2D) echocardiography with the assessment of the maximal left ventricular wall thickness in diastole (MWT), left atrial diameter (LAD), and maximal provocable left ventricular outflow tract (LVOT) gradient [1]. For the disease history, we check the following binary variables: syncope and family history of sudden death [1]. Finally, we include into the calculator the age of patients [1].

### 2.2. Second Model of Risk Calculation (All ECG Holter − Current + Previous ECG Holter)

Every patient had at least 3 times 24-hour ECG Holter recordings during life. One ECG Holter is defined as current (simultaneous) with biomarker sampling, and the remaining 2 or more recordings took place in past history. Presence/absence of NsVT was assessed by summing the data of all Holter (previous and current). The remaining parameters used in calculation were identical as in the first model.

The study protocol was approved by a local institutional review board (Komisja Bioetyki Jagiellonian University).

### 2.3. Statistical Analysis

Normally distributed continuous variables were presented as mean ± SD. Differences between two groups were assessed using independent *t-*test. Categorical variables were assessed using the Fisher exact test and expressed as numbers (percentages). A *P* value of less than 0.05 was considered significant.

## 3. Results

The baseline characteristics of HCM patients are displayed in Table 1.

Hs-TnI was detected in all HCM patients and patients with abnormal level > 19.5 ng/L were defined as positive troponin subgroup; nonelevated hs-TnI subgroup consisted of negative troponin subgroup. After NT-proBNP measurement, only 3 patients have a normal concentration < 125 pg/mL; thus, subgroup division has been created by a median.

In comparison between 19 patients (hs-TnI positive) versus 27 patients (hs-TnI negative), the calculated 5-year percentage risk of SCD in HCM was significantly greater, both in the first and in the second models (Table 2). In the second comparison between higher NT-proBNP versus lower NT-proBNP subgroups, the calculated 5-year percentage risk of SCD in HCM was not significantly greater in the first model as well as in the second model (Table 2).

## 4. Discussion

In the current study, patients with HCM and positive hs-TnI test have a higher risk of SCD estimated according to SCD calculator recommended by the ESC Guidelines 2014 than patients with negative hs-TnI test. Level of NT-proBNP is not associated with the calculated 5-year risk of SCD (stratified by calculator).

### 4.1. Clinical Application of Biomarkers in HCM

In a recent review paper, authors ask the question: NT-proBNP versus troponin: is one better than the other [2]. Although both biomarkers correlate with indices of HCM disease progression, BNP may be a more sensitive indicator of left ventricular hypertrophy than troponin. It was documented that the wall thickness threshold was lower for BNP elevation than for TnI elevation [14]. Moreover, it has also stronger predictors of hemodynamic parameters and clinical symptoms than troponin. Although a correlation between elevated troponin and elevated BNP has been demonstrated [14, 15], it is not a consistent finding [8, 14].

Before our study, a strategy for application of these biomarkers to risk stratification has not yet been investigated. These biomarkers may be most useful when risk markers of SCD indicate intermediate or indeterminate risk.

The impact of stress echocardiography in HCM is limited by lack of standardization and outcome data. ECS guidelines recommend stress echo solely for evaluation of LVOT [3]. However, large-scale registry data show that stress echocardiography positivity for ischemic criteria (such as new wall motion abnormalities and coronary flow velocity reserve) rather than inducible gradients predicts adverse outcome in HCM [4]. In a large study [3], mortality was predicted using criteria for detecting ischemia on stress echocardiography. The investigators proposed that stress echocardiography has a signficant prognostic role in patients with HCM, with ischemic endpoints showing a greater predictive accuracy than hemodynamic endpoints [3].

In a recent review by McCarthy et al. [16], the utility of hs-Tn assessment in arrhythmic disease is only at the initial stage of investigations, but it has been postulated as a valuable screening marker for patients with HCM at high risk of SCD.

Regular training exercise (e.g., fitness activity) has recently been recommended for selected patients with HCM [17]. Based on current observation on the association between tachycardia and elevated troponin level in patients with HCM [18] and phenomenon of troponin release by exercise [19], we suggest that any exercise stress test in HCM patients (performed either for training or diagnostic purposes) should be controlled by troponin level measurements before exercise and 6 and 12 hours after the exercise. Especially predisposed to high LVOT gradient are HCM women > 50 years of age, due to smaller LV cavity size [20]. This subgroup of HCM patients may be particularly at risk to develop high gradient at peak/post exercise period resulting into increased calculated risk in calculator.

Our study has several limitations. First, our study group may appear too small to definitely rule out association between NT-proBNP and SCD HCM risk score. Secondly, the current pharmacological treatment was maintained, and particularly, *β*-blockers were not withdrawn. In our pilot study, we aimed to make the observation on the correlation between hs-TnI release and timely synchronized findings on ECG Holter and resting/stress echocardiography.

Our preliminary study showed that *β*-blocker withdrawal might not be safe in troponin-positive subgroup of patients. In future studies, we will attempt to increase the dose and use only one type of a *β*-blocker to decrease ischemia burden.

We decided to measure hs-TnI levels only once because our pilot study was conducted in an outpatient setting. The optimal protocol, that is, 48-hour profile of troponin measurement with the assessment of troponin with echocardiographic examination every 8 hours, and 48 hours Holter monitoring (recommended by the ESC Guidelines), would require the in-hospital setting for the study and would be more costly. Moreover, only an outpatient-based study provides an opportunity to assess the heart rate during common daily physical activity of patients. At first look, the study by Kubo et al. [5] seems similar to our study; however, we have found essential differences from our investigation in many important points.

- Kubo et al. (in pre-era of the ESC Guidelines from 2014, authors did not use calculator risk factors for SCD) defined their study as analysis of combined cardiovascular events (with SCD episode [the most fatal end point] only in 4 patients—such number seems to be too small for proper statistical analysis). Our study was not follow-up designed, but focused on relationship between biomarker levels and calculated 5-year percentage risk score only for SCD in HCM. SDC is the most fatal, but an easy preventable complication of HCM (by ICD).
- Morphologic/prognostic differences are also important (in Kubo et al., patients had less predisposition for SCD: a benign apical variant 28% versus 0% in our group). The benign morphologic pattern in Kubo et al. paper seems to correspond to the low number of SCD.
- In Kubo et al., LVOT gradient was assessed in binary analysis < 30 mmHg or ≥30 mmHg and only in resting conditions. In contrast, we have assessed LVOTG more precisely as a continuous variable, measured both at rest and after provocation (the provocable LVOTG is absolutely needed to measure risk of SCD by ESC calculator).
- Kubo et al. did not analyze nsVT in ECG-Holter (which is absolutely needed to measure the risk of SCD by calculator); moreover, nsVT assessment is needed also in the American Guideline from 2011 for risk stratification of SDC. Thus, in a paper by Kubo et al., the lack of ECG Holter analysis is a serious limitation.
- Kubo et al. did not describe the time period between blood sampling for biomarkers and measurement of echo parameters (nsVT in Holter was not studied). In our study, the time synchrony between echo/Holter and hs-TnI measurement was defined.
- Kubo et al. did not analyze NT-proBNP, but only hs-Tn. Our study provides more information about two important biomarker sampling simultaneously with echocardiographic and ECG Holter measurements.

## 5. Conclusions

Patients with HCM and positive hs-TnI test have higher risk of SCD estimated according to SCD calculator recommended by the ESC Guidelines 2014 than patients with negative hs-TnI test.

### 5.1. Clinical Perspective

These findings suggest that hs-Tn may be useful as an additional biomarker for better risk stratification in HCM. Additionally, we have postulated to monitor also the biomarkers of endothelial dysfunction (impaired endothelium-dependent vasodilatation) [21].

## Figures and Tables

**Table 1:** Baseline characteristics of the patients.

NYHA class	2.3 ± 0.6
CCS class	1.5 ± 0.4
Syncope (*n*)	15
Sudden death in family history (*n*)	15
NSVT in current Holter (*n*)	11
Creatinine, *μ*g/L	82.3 ± 11.6
Maximum LV thickness, mm	22.5 ± 4.2
Resting LVOT gradient, ≥30 mm Hg (*n*)	8
Provocable LVOT gradient, ≥30 mm Hg (*n*)	17
Left atrial diameter, mm, mean (SD)	4.83 ± 0.81
Drugs with negative chronotropic properties (*n*)	
*β*-Blocker	37
Verapamil	5
None	4

CCS: Canadian Cardiovascular Society; LVOT: left ventricular outflow tract; LV: left ventricular; NSVT: nonsustained ventricular tachycardia; NYHA: New York Heart Association.

**Table 2:** Comparison between subgroups of hs-TnI positive versus negative and also between subgroup of lower versus higher NT-proBNT concentration (NS: nonsignificant).

First model—current Holter			
	Hs-TnI negative *n* = 27	Hs-TnI positive *n* = 19	
5-year SCD risk in HCM	3.81 ± 3.23%	6.38 ± 4.17%	*P* < 0.05
	Lower NT-proBNP *n* = 23	Higher NT-proBNP *n* = 23	
5-year SCD risk in HCM	4.14 ± 4.18%	5.18 ± 3.63%	NS

Second model—all Holter			
	Hs-TnI negative *n* = 27	Hs-TnI positive *n* = 19	
5-year SCD risk in HCM	4.25 ± 4.20%	6.90 ± 3.99%	*P* < 0.05
	Lower NT-proBNP *n* = 23	Higher NT-proBNP *n* = 23	
5-year SCD risk in HCM	4.40 ± 3.62%	6.29 ± 4.18%	NS
